# Transferable IncX3 plasmid harboring *bla*_NDM-1_, *ble*_MBL_, and *aph(3’)*-VI genes from Klebsiella pneumoniae conferring phenotypic carbapenem resistance in* E. coli*

**DOI:** 10.1007/s11033-023-08401-9

**Published:** 2023-04-20

**Authors:** Ann A. Elshamy, Sarra E. Saleh, Khaled M. Aboshanab, Mohammad M. Aboulwafa, Nadia A. Hassouna

**Affiliations:** 1 Department of Microbiology and Immunology, Faculty of Pharmacy, Ain Shams University, Organization of African Unity St, P.O. Box 11566, Cairo, 11566 Egypt; 2 Faculty of Pharmacy, King Salman International University, South Sinai, Ras-Sedr, Egypt

**Keywords:** NDM, Carbapenem resistance, CRE, APH(3’)-VI, Plasmid, Antimicrobial resistance

## Abstract

**Background:**

The dissemination of carbapenem resistance via carbapenemases, such as the metallo-β-lactamase NDM, among *Enterobacterales* poses a public health threat. The aim of this study was to characterize a plasmid carrying the *bla*_NDM-1_ gene, which was extracted from a clinical *Klebsiella pneumoniae* uropathogen from an Egyptian patient suffering from a urinary tract infection.

**Methods and results:**

The recovered plasmid was transformed into competent *E. coli* DH5α which acquired phenotypic resistance to cefoxitin, ceftazidime, and ampicillin/sulbactam, and intermediate sensitivity to ceftriaxone and imipenem (a carbapenem). Whole plasmid sequencing was performed on the extracted plasmid using the DNBSEQ™ platform. The obtained forward and reverse reads were assembled into contigs using the PRINSEQ and PLACNETw web tools. The obtained contigs were uploaded to PlasmidFinder and ResFinder for *in silico* plasmid typing and detection of antimicrobial resistance genes, respectively. The final consensus sequence was obtained using the Staden Package software. The plasmid (pNDMKP37, NCBI accession OK623716.1) was typed as an IncX3 plasmid with a size of 46,160 bp and harbored the antibiotic resistance genes *bla*_NDM-1_, *ble*_MBL_, and *aph(3’)-VI*. The plasmid also carried mobile genetic elements involved in the dissemination of antimicrobial resistance including insertion sequences IS*30*, IS*630*, and IS*26*.

**Conclusions:**

This is Egypt’s first report of a transmissible plasmid co-harboring *bla*_NDM-1_ and *aph*(3’*)-VI* genes. Moreover, the respective plasmid is of great medical concern as it has caused the horizontal transmission of multidrug-resistant phenotypes to the transformant. Therefore, new guidelines should be implemented for the rational use of broad-spectrum antibiotics, particularly carbapenems.

**Supplementary Information:**

The online version contains supplementary material available at 10.1007/s11033-023-08401-9.

## Introduction

Antimicrobial resistance (AMR) has been on the rise globally over the last decade, in addition to a shortage of functional antimicrobials, and a lack of novel ones. Such resistance has a major impact on treatment outcomes, resulting in higher antimicrobial costs, longer hospital stays, greater hospital expenses, and higher fatality rates [1]. In an effort to overcome the increasing rates of resistance to penicillins, cephalosporins, aminoglycosides, and fluoroquinolones, the use of carbapenems has increased in recent years [2, 3]. Regrettably, the extensive usage of carbapenems has led to the development of carbapenem-resistance (CR) particularly, in Gram-negative bacteria (GNB) such as *Enterobacterales*, *Acinetobacter* spp., and *Pseudomonas* spp. This has resulted in a global public health crisis due to the rapid spread of CR and the scarcity of novel antimicrobials [4–6].

The World Health Organization (WHO) announced in 2017 a global priority pathogens list for which new antibiotics are urgently needed, among which carbapenem-resistant *Enterobacterales* (CRE) that are co-resistant to 3rd generation cephalosporins were listed as critical priority pathogens [7]. Infections caused by CRE pathogens have caused substantial morbidity and mortality, and are considered as a rising healthcare threat [8, 9]. Carbapenemase-producing *K. pneumoniae* represents the fastest-growing threat to antibiotic resistance in terms of morbidity and mortality [10–12].

Among the different mechanisms of CR, carbapenemases represent the greatest threat in terms of AMR spread, due to their ability to inactivate most β-lactams, and the fact that they are encoded by genes conferred by mobile genetic elements (MGEs) such as transposons, insertion sequences, or plasmids, which are capable of interspecies and intraspecies horizontal transfer [13, 14]. Plasmids are extrachromosomal, self-replicating DNA units that encode nonessential but usually useful traits for their host. Several copies of one or more plasmids may be present within a bacterial cell [15]. Many plasmids encode genes for resistance to antimicrobial agents and heavy metals, virulence factors, production of toxins, attachment to intestinal mucosa, and for new pathways of degradation [16]. Acquisition of such plasmids enables the host bacterium to adapt to environmental changes, such as exposure to antibiotics, rapidly and effectively [17]. Plasmid replicon typing was established in order to facilitate their identification and study [18].

The New Delhi metallo-β-lactamase (NDM), a carbapenemase that belongs to Ambler class B β-lactamases, was discovered in 2008 when a Swedish patient who had traveled to New Delhi, India, acquired a urinary tract infection caused by a carbapenem-resistant *K. pneumoniae.* The strain isolated from his urine was a metallo-β-lactamase (MBL) producer but was negative for previously known MBL genes. This led to the discovery of the *bla*_NDM-1_ gene [19]. It was later revealed that NDM β-lactamases confer resistance to almost all β-lactam antimicrobials (except aztreonam), including carbapenems which are often used as last-resort treatment options for multidrug-resistant (MDR) and extended-spectrum β-lactamase (ESBL) producers-associated infections [20, 21].

Genes coding for NDM enzymes are highly transmissible as they are often located on plasmids harboring several other antimicrobial resistance determinants, thus, NDM-producing bacteria are often resistant to aminoglycosides and fluoroquinolones, a fact which has posed challenges in the clinical treatment of infections caused by CRE [20, 22, 23].

In this study, we report the sequence of a transmissible IncX3 plasmid carrying a carbapenemase resistance gene (*bla*_NDM-1_), bleomycin resistance gene (*ble*_MBL_), and aminoglycoside resistance gene (*aph(3’)-VI*), which was extracted from a carbapenem-resistant *K. pneumoniae* clinical isolate recovered from the urine of an Egyptian patient suffering from a urinary tract infection.

## Materials and methods

### Collection of the clinical isolate

Carbapenem-resistant *K. pneumoniae* isolate (code: 37.AK) was obtained from El-Demerdash Tertiary Care Hospital’s Microbiology laboratory in Cairo, Egypt. According to the hospital’s records, the isolate was recovered from the urine sample of a male patient admitted to the hospital with significant bacteriuria (uropathogens > 10^5^ cfu/ml) in May 2020. The identification of the isolate was carried out using phenotypic and cultural characteristics [24]. Identification of the isolate was further confirmed by using the commercially available MIKROLATEST^®^ ID Kit ENTEROtest 24 N (Erba Lachema, The Czech Republic) [25] following the manufacturer’s instructions. The patient had no history of international travel. Written and oral informed consents were attained from the patient. The study was approved by the Ethics Committee of the Faculty of Pharmacy Ain Shams University (EN-REC-ASU-2019-98) and was in accordance with the Declaration of Helsinki.

### Antimicrobial susceptibility testing

The Kirby-Bauer disk diffusion test was done [26] using 17 antimicrobials including meropenem (10 µg), imipenem (10 µg), doripenem (10 µg), ertapenem (10 µg), ampicillin/sulbactam (20 µg), amoxicillin/clavulanic acid (30 µg), ceftriaxone (30 µg), cefoxitin (30 µg), ceftazidime (30 µg), cefepime (30 µg), ciprofloxacin (5 µg), levofloxacin (5 µg), amikacin (30 µg), gentamicin (10 µg), trimethoprim/sulfamethoxazole (25 µg), fosfomycin (200 µg), and nitrofurantoin (300 µg). After incubation, the inhibition zone diameters were measured and susceptibility was interpreted by referring to Clinical and Laboratory Standards Institute (CLSI) guidelines 2020 [27]. The reference strain *E. coli* ATCC^®^ 25,922 was used as control.

### Detection of carbapenemase production

Blue-Carba test was performed as described by Pires et al. for the detection of carbapenemases directly from bacterial cultures [28]. The modified carbapenem inactivation method (mCIM) was also used to detect the production of carbapenemases as recommended by the CLSI 2020 [27]. The reference strain *E. coli* ATCC^®^ 25,922 was used as control.

### Amplification of some plasmid-encoded carbapenemase genes

The plasmid DNA was extracted using the GeneJet Plasmid Miniprep Kit (catalog number: K0502 Thermo Fisher Scientific, Lithuania). The *bla*_NDM_, *bla*_KPC_, *bla*_OXA-48,_*bla*_VIM_, and *bla*_IMP_ carbapenemase genes were amplified using the plasmid extract as a template for PCR and the appropriate primers synthesized by Invitrogen^®^ (Thermo Fisher Scientific, UK), and DreamTaq™ Green PCR Master Mix (Thermo Fisher Scientific, Lithuania). Gel electrophoresis was used to analyze the PCR products as previously reported [29]. Table 1 shows the primers used for PCR amplification of the tested carbapenemase-encoding genes, their annealing temperatures, and the expected product sizes of the tested genes.

**Table 1:** The primers used for PCR amplification of the tested carbapenemase-encoding genes

Gene	Primer	Primer sequence (5ʹ ◊ 3ʹ)	Expected PCR product size (bp)	T_a_ (°C)	References
*bla*_NDM_	P_f_	GGTTTGGCGATCTGGTTTTC	621	50	[41, 42]
	P_r_	CGGAATGGCTCATCACGAT			
*bla*_KPC_	P_f_	TGTCACTGTATCGCCGTC	1011	50	[40]
	P_r_	CTCAGTGCTCTACAGAAAACC			
*bla*_OXA-48_	P_f_	GCGTGGTTAAGGATGAACAC	438	50	[40, 42]
	P_r_	CATCAAGTTCAACCCAACCG			
*bla*_VIM_	P_f_	TCTACATGACCGCGTCTGTC	748	50	[43]
	P_r_	TGTGCTTTGACAACGTTCGC			
*bla*_IMP_	P_f_	CTACCGCAGCAGAGTCTTTG	587	50	[44]
	P_r_	AACCAGTTTTGCCTTACCAT			

**Notes**: *bla*_NDM_, *bla*_KPC_, *bla*_OXA-48,_*bla*_VIM_, and *bla*_IMP_ genes code for NDM, KPC, OXA-48-like, VIM, and IMP carbapenemases, respectively. Abbreviations: T_a_, annealing temperature; P_f_, forward primer; P_r_, reverse primer

### Transformation

Chemical transformation was performed according to the protocol described by Sambrook and Russell [29]. *E. coli* DH5α is a standard susceptible strain free from antimicrobial resistance genes. The preparation of competent *E. coli* DH5α cells was carried out via the modified Hanahan method [30]. The plasmid extract was used to transform the competent *E. coli* DH5α. The transformant was cultured on an LB/ampicillin agar plate at a concentration of 100 µg/ml. Untransformed *E.coli* DH5α was used as a negative control. Blue-Carba test, mCIM test, and antimicrobial susceptibility testing of the transformant were carried out, as well as the determination of the minimum inhibitory concentrations (MICs) of meropenem and imipenem by broth microdilution method following CLSI guidelines [27, 31]. The EDTA-modified carbapenem inactivation method (eCIM) is a phenotypic test used to differentiate MBLs (e.g. NDM, VIM, and IMP carbapenemases) from serine carbapenemases (e.g. OXA-48, and KPC carbapenemases) in *Enterobacterales* isolates showing positive mCIM test results. This test was carried out and interpreted as mentioned in the CLSI guidelines [27] to determine the type of carbapenemase enzyme produced by the transformant. The plasmid DNA was then extracted from the transformant (TS37.AK) to be used as a template for PCR amplification for confirming the presence of carbapenemase genes on the transformed plasmid using the primers listed in Table 1.

### Plasmid sequencing and bioinformatic analysis

The transformant was sub-cultured 2 successive times on LB/ampicillin (100 µg/ml), followed by a third time on LB/meropenem (4 µg/ml), to increase the copy number of the plasmid by positive pressure. The plasmid DNA was then extracted from the transformant, assigned the name pNDMKP37, and was sent for whole plasmid sequencing at BGI TECH SOLUTIONS (HONGKONG) CO., LIMITED (Tai Po, Hong Kong) using DNBSEQ™ platform with PE100. The obtained clean reads were assembled into contigs using the PReprocessing and INformation of SEQuences (PRINSEQ) v0.20.4 (http://prinseq.sourceforge.net/) (accessed on 15 December 2022) and PLACNETw (https://castillo.dicom.unican.es/upload/) (accessed on 15 December 2022) web tools [32, 33]. The obtained contigs were uploaded to PlasmidFinder 2.1 (https://cge.cbs.dtu.dk/services/PlasmidFinder/) (accessed on 15 December 2022) for *in silico* plasmid typing. ResFinder 4.1 (https://cge.cbs.dtu.dk/services/ResFinder/) (accessed on 15 December 2022) and the Comprehensive Antibiotic Resistance Database (CARD) (https://card.mcmaster.ca/)(accessed on 15 December 2022) were used to detect antimicrobial resistance genes. The consensus sequence was finally assembled using the Staden Package Gap4 software v2.0.0b11 (http://staden.sourceforge.net/)(accessed on 15) [34]. The constructed plasmid was automatically annotated via Bacterial and Viral Bioinformatics Resource Center (BV-BRC) (https://www.bv-brc.org/) (accessed on 17 December 2022) followed by manual inspection for confirmation and correction using ORFfinder (https://www.ncbi.nlm.nih.gov/orffinder/)(accessed on 17 December 2022) and BLAST tools (https://blast.ncbi.nlm.nih.gov/Blast.cgi) (accessed on 17 December 2022).

The plasmid sequence was uploaded to the plasmid database PLSDB (https://ccb-microbe.cs.uni-saarland.de/plsdb/) (accessed on 3 December 2022) to search the database for plasmids with nucleotide sequences similar to our plasmid (pNDMKP37). The search strategy used was “Mash dist.” (for long sequences e.g., contigs or long reads), and the parameters were maximum *p*-value of 0.05 and maximum distance of 0.05. The creation of the circular image and comparison with other reported similar plasmids were performed using the BLAST Ring Image Generator (BRIG) tool v0.95 (https://sourceforge.net/projects/brig/)(accessed on 3 December 2022) [35].

### Nucleotide sequence accession number and data availability

The plasmid pNDMKP37 sequence project has been deposited in the GenBank under BioProject PRJNA878540, sample number SAMN30732688, and with the plasmid sequence accession number OK623716.1. The Sequence Read Archive (SRA) data are available from GenBank under the accession number SRR21492341.

## Results

### Antimicrobial susceptibility, carbaenemase-production, and PCR amplification of carbapenemase-encoding genes

The results of antimicrobial susceptibility, Blue-Carba test, mCIM, and PCR amplification of carbapenemase-encoding genes of the carbapenem-resistant *K. pneumoniae* clinical isolate (37.AK) were previously published [36]. In terms of antimicrobial susceptibility to carbapenems, the isolate was resistant to meropenem, imipenem, and ertapenem, and was susceptible to doripenem.

### Transformation

The transformant (TS37.AK) showed growth on an LB/ampicillin agar plate (100 µg/ml), indicating the successful transformation of a plasmid harboring at least one β-lactam resistance gene. Antimicrobial susceptibility results showed that the transformant (TS37.AK) acquired a new antimicrobial resistance profile compared to the untransformed *E. coli* DH5α, where this plasmid conferred phenotypic resistance to ceftazidime, cefoxitin, and ampicillin/sulbactam, and intermediate sensitivity to ceftriaxone and imipenem in the transformant. The transformant remained susceptible to ertapenem and doripenem. The MICs of meropenem and imipenem against the transformant were 0.5 µg/ml (susceptible) and 2 µg/ml (intermediate sensitivity), respectively. The results of Blue-Carba and mCIM tests were positive for the transformant indicating carbapenemase-production. The results of eCIM test confirmed that the transformant was an MBL producer, where the carbapenemase inhibition test with EDTA showed a complete inhibition of the carbapenemase activity indicating that the transformant was a sole MBL producer. The phenotypic characteristics of the transformant (TS37.AK), the respective parent *K. pneumoniae* clinical isolate (37.AK), and the untransformed *E. coli* DH5α were previously published [36] and are demonstrated in Table 2. After the plasmid DNA was extracted from the transformant, the results of PCR amplification of carbapenemase-encoding genes confirmed that the transformant was carrying *bla*_OXA-48_ and *bla*_NDM_ genes (Figures S1 and S2).

**Table 2:** The results of antimicrobial susceptibility testing and phenotypic carbapenemase production of the transformant TS37.AK compared to the respective parent clinical isolate *K. pneumoniae* 37.AK and the untransformed *E. coli* DH5α

	Parent clinical isolate *K. pneumoniae* 37.AK	Transformant TS37.AK	Untransformed *E. coli* DH5α
Antimicrobial susceptibility testing			
Antimicrobial agent	Phenotypic resistance profile		
Meropenem (10 µg)	**R**	S	S
Imipenem (10 µg)	**R**	**I**	S
Doripenem (10 µg)	S	S	S
Ertapenem (10 µg)	**R**	S	S
Ampicillin/sulbactam (20 µg)	**R**	**R**	S
Amoxicillin/clavulanic acid (30 µg)	S	S	S
Cefepime (30 µg)	**R**	S	S
Ceftriaxone (30 µg)	**R**	**I**	S
Ceftazidime (30 µg)	**R**	**R**	S
Cefoxitin (30 µg)	**R**	**R**	S
Gentamicin (10 µg)	**R**	S	S
Amikacin (30 µg)	**R**	S	S
Levofloxacin (5 µg)	**R**	S	S
Ciprofloxacin (5 µg)	**R**	S	S
Trimethoprim/sulfamethoxazole (25 µg)	**R**	S	S
Fosfomycin (200 µg)	**R**	S	S
Nitrofurantoin (300 µg)	S	S	S
**Phenotypic detection of carbapenemase production**			
Blue-Carba test	+ve	+ve	-ve
Modified carbapenem inactivation method (mCIM)	+ve	+ve	-ve

**Abbreviations:** S, susceptible; R, resistant; I, intermediate sensitivity.

### Plasmid sequencing and bioinformatic analysis

## The assembled consensus sequences

**Fig. 1:**
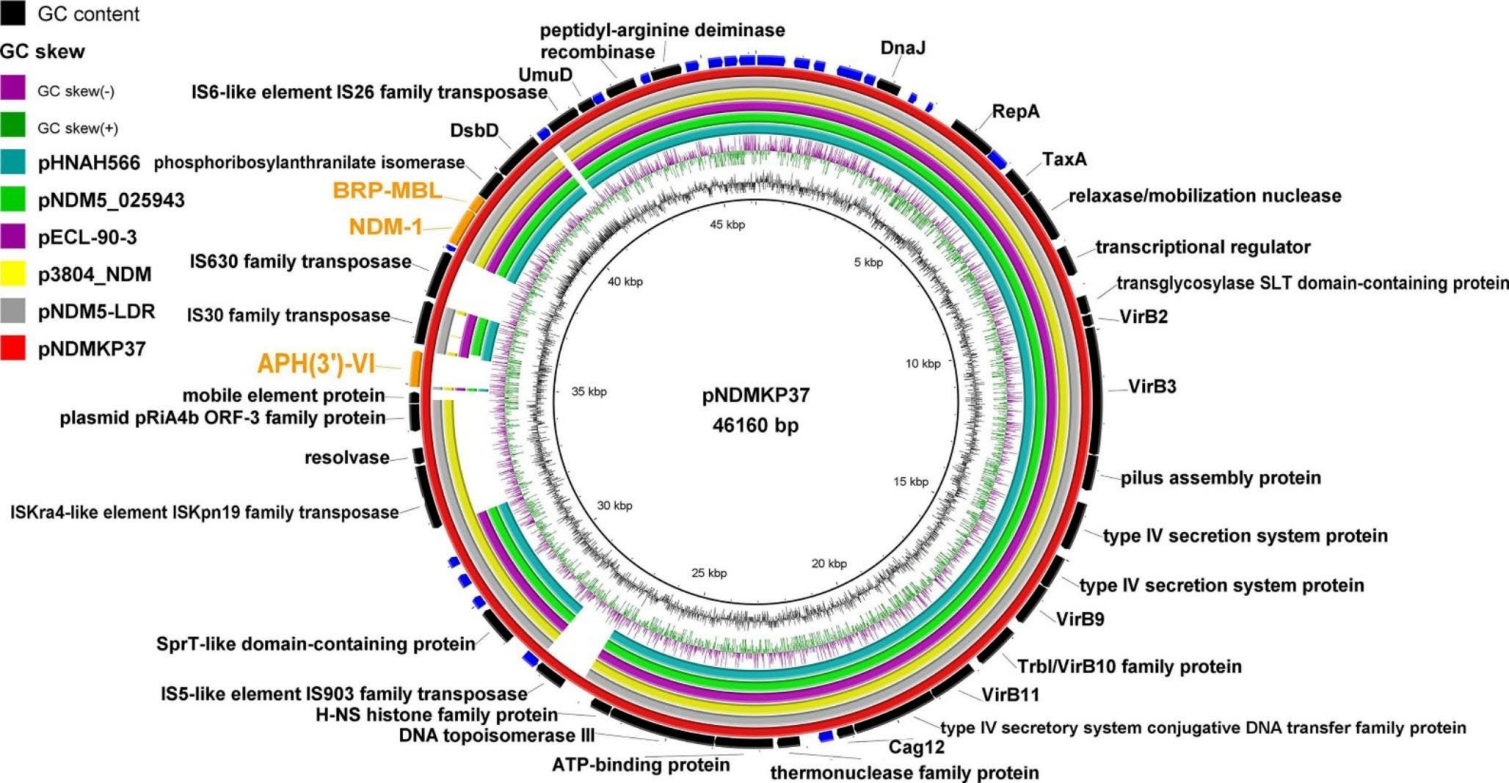
The circular map of the NDM-positive plasmid pNDMKP37 (*K. pneumoniae* strain 37.AK; AC, OK623716.1) compared to other reported similar plasmids from different *Enterobacterales* strains, including pNDM-EC36 (*E. coli* strain EC36; AC, NZ_MG591703.1), pNDM5_020046 (*K. pneumoniae* subsp. *pneumoniae* strain SCKP020046; AC, CP028781.2), p3804_NDM (*Enterobacter hormaechei* strain 3804; AC, CP064660.1), pRIVM_C018652_2 (*K. pneumoniae* strain RIVM_C018652; AC, CP068835.1), and pNDM5-LDR (*K. pneumoniae* strain LDR; AC, MK308632.1). The red ring represents the plasmid used as a reference for the alignment (pNDMKP37); the size of the reference is indicated in the center of the panel. The genes coding for APH (3ʹ)-VI, NDM-1, and BRP_MBL_ proteins are marked in orange in the outer ring, and hypothetical proteins are marked in blue. AC, GenBank accession code

Clean forward and reverse sequence reads were assembled into 6 contigs. The plasmid belonged to the IncX3 plasmid incompatibility group and was found to carry 3 resistance genes, namely *bla*_NDM-1_ coding for NDM-1 carbapenemase, *ble*_MBL_ coding for bleomycin resistance protein BRP_MBL_, and *aph(3’)-VI* gene coding for APH(3’)-VI (aminoglycoside 3′- phosphotransferase). The final obtained consensus sequence length was 46,160 bp with 46.28% G + C content. The plasmid contained 58 open reading frames (ORFs) (37 genes of known functions and 21 genes of hypothetical proteins, Fig. 1). The insertion sequence IS*30* family transposase (98.98% similarity to IS*Aba125*) was found upstream of *bla*_NDM-1_, and bleomycin resistance gene, *ble*_MBL_, was located downstream. The genes *dsb* and *trpF* were found downstream of *ble*_MBL_, all of which are common genetic contexts of *bla*_NDM_ gene variants [37]. Other MGEs were found in the genetic environment of *bla*_NDM-1_, including the insertion sequences IS*630* and IS*26*, which can promote the mobilization of *bla*_NDM-1_ between plasmids or chromosomes.

## Discussion

The most effective antimicrobials for treating infections caused by MDR bacteria are carbapenems. This class of antimicrobials exhibits a wide spectrum of activity against both Gram-negative and Gram-positive bacteria. The overuse of carbapenems in many nations has accelerated the emergence of carbapenem resistance, resulting in a worldwide public health crisis [38]. Increasing numbers of *Enterobacterales* (especially *Klebsiella* spp.) and lactose non-fermenters are acquiring and producing carbapenemases. NDMs, which confer resistance to carbapenems and other β-lactam antibiotics, have been increasingly reported across the world since their first report.

In this study we report an IncX3 transmissible plasmid carrying 3 resistance genes: the carbapenemase resistance gene (*bla*_NDM-1_) coding for NDM-1 carbapenemase, bleomycin resistance gene (*ble*_MBL_) coding for bleomycin resistance protein BRP_MBL_, and aminoglycoside resistance gene (*aph(3’)-VI*) coding for aminoglycoside 3′- phosphotransferase APH(3’)-VI. The plasmid was extracted from a carbapenem-resistant *K. pneumoniae* clinical isolate recovered from the urine of an Egyptian patient suffering from a urinary tract infection. In our previous study involving the same transformant (TS37.AK), the PCR amplification results of the transferable plasmid revealed the presence of genes coding for carbapenemases in the plasmid extract. However, after whole plasmid sequencing in the current study, the sequencing data confirmed that this plasmid carried the *bla*_NDM-1_ gene coding for NDM-1 carbapenemase, as well as other resistance determinants namely the *ble*_MBL_ gene coding for bleomycin resistance protein BRP_MBL_, and *aph(3’)-VI* gene coding for APH(3’)-VI (aminoglycoside 3′- phosphotransferase).

Generally, the *bla*_NDM_ gene variants share two common features pertaining to their genetic environment: the bleomycin resistance gene (*ble*_MBL_) is always downstream of *bla*_NDM_, and the IS*Aba125* insertion sequence (either intact or truncated) is always upstream. Further downstream of *ble*_MBL_, there is often a set of several genes, including *trpF* (which encodes a phosphoribosylanthranilate isomerase), and *dsb* (which encodes a twin-arginine translocation pathway signal sequence domain protein) [37]. This was evident in the sequence of the plasmid in the present study (pNDMKP37), where *ble*_MBL_ was located downstream of *bla*_NDM-1_, and the insertion sequence IS*30* family transposase (98.98% similarity to IS*Aba125*) was found upstream. Additionally, the genes *trpF* and *dsb* were found downstream of *ble*_MBL_. Other MGEs were found in the genetic context of *bla*_NDM-1_, including insertion sequences IS*630* and IS*26*, which can promote the mobilization of *bla*_NDM-1_ between plasmids or chromosomes.

As for the *bla*_OXA-48_ gene, it is generally associated with an upstream IS*1999* element, and a downstream composite transposon Tn*1999* [39]. Other *bla*_OXA-48-_like genes have been found on plasmids in association with other insertion sequences, for example the *bla*_OXA-163_ gene was located downstream of an IS*Ecl4* element, and *bla*_OXA-181_, *bla*_OXA-204_, and *bla*_OXA-232_ genes were associated with IS*Ecp1* elements [39]. In our study, although *bla*_OXA-48_ gene was detected by PCR in the plasmid extract of the transformant (TS37.AK), neither the gene nor its usual genetic context were detected on the sequenced plasmid. To confirm that the *bla*_OXA-48_ gene not being in the plasmid was not a bioinformatics processing error, whole genome DNA templates of the transformant were prepared as described by Doyle et al. [40], and the supernatant was used as a template for PCR amplification of *bla*_OXA-48_ and *bla*_NDM_ genes. This was done to detect the possibility that the *bla*_OXA-48_ could be carried on a MGE that was not incorporated in the sequenced plasmid and was instead chromosomally incorporated. The PCR results confirmed the presence of the *bla*_NDM_ gene and the absence of the *bla*_OXA-48_ gene from the whole genome of the transformant, although the *bla*_OXA-48_ gene was previously detected in the initial plasmid extract of the transformant by PCR and subsequent sanger sequencing of the resulting amplicon [36]. It was thus speculated that the MGE carrying the *bla*_OXA-48_ gene might have been lost from our plasmid after repeated subculturing of the transformant, which could be proved by whole genome sequencing of the clinical isolate and the transformant.

**Fig. 2:**
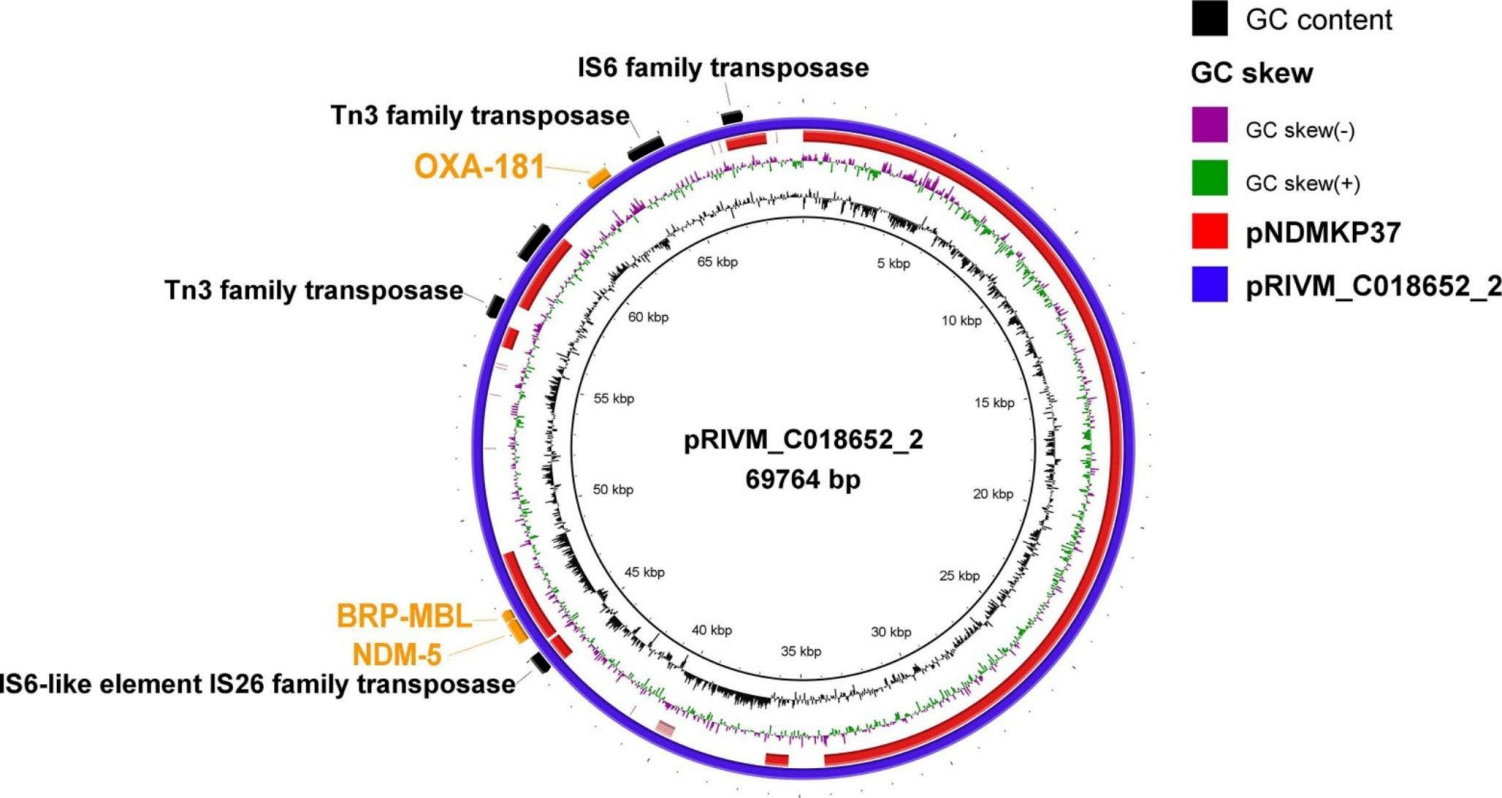
The circular map of the NDM-positive plasmid pNDMKP37 (*K. pneumoniae* strain 37.AK; AC, OK623716.1) compared to the OXA-181 and NDM-positive plasmid (K. *pneumoniae* strain RIVM_C018652; AC, NZ_CP068835.1). The blue ring represents the plasmid used as a reference for the alignment (pRIVM_C018652_2); the size of the reference is indicated in the center of the panel. The genes on plasmid pRIVM_C018652_2 coding for NDM-5, BRP_MBL_, and OXA-181 proteins are marked in orange in the outer ring. Transposases are marked in black. AC, GenBank accession code

The PLSDB plasmid database was searched for plasmids having nucleotide sequences similar to our plasmid (pNDMKP37). The results of searching PLSDB database showed 397 hits, of which 250 (62.97%) plasmids carried *bla*_NDM_ gene variants as follows: 63 plasmids carried the *bla*_NDM-1_, 9 carried the *bla*_NDM-4_, 143 carried *bla*_NDM-5_, 1 harbored *bla*_NDM-6_, 27 carried the *bla*_NDM-7_, 1 carried *bla*_NDM-11_, 1 harbored *bla*_NDM-13_, 1 harbored *bla*_NDM-17_, 2 harbored *bla*_NDM-19_, 1 carried *bla*_NDM-20_, and 1 carried *bla*_NDM-21_ variant. It was noticed that similar to our plasmid (pNDMKP37), 246/250 plasmids (98.4%) carrying the *bla*_NDM_ gene variants also harbored the bleomycin resistance determinant *ble*. Seventy of the similar plasmids (17.6%) carried the *bla*_OXA-181_ resistance determinant, and only 1 of which (plasmid pRIVM_C018652_2, accession no: NZ_CP068835.1) carried both *bla*_NDM-5_ (a *bla*_NDM_ variant), and *bla*_OXA-181_ (a *bla*_OXA-48_ variant) genes together. This plasmid was extracted from a *K. pneumoniae* isolate as well. When the nucleotide sequences of both plasmids were compared, it was found that the *bla*_OXA-181_ gene in plasmid pRIVM_C018652_2 was carried on a MGE (as shown in Fig. 2) whose sequence was missing from our plasmid (pNDMKP37). It was also noted that the size of pRIVM_C018652_2 (69,764 bp) was larger than that of pNDMKP37 (46,160 bp).

It was noticed that the parent *K. pneumoniae* clinical isolate (37.AK) was resistant to aminoglycosides (gentamicin and amikacin) and quinolones (ciprofloxacin and levofloxacin), however, such resistance was not conferred to the transformant by the transformed plasmid. This is probably because resistance mechanisms to such antimicrobial agents was chromosomally-mediated in the parent clinical isolate.

## Conclusions

In this study, a transmissible plasmid co-harboring *bla*_NDM-1_ and *aph(3’)-VI* genes was detected in a *K. pneumoniae* clinical isolate in Egypt. The plasmid (pNDMKP37) was typed as an IncX3 plasmid with a size of 46,160 bp and harbored antibiotic resistance genes against carbapenems (*bla*_NDM-1_), bleomycin (*ble*_MBL_), and aminoglycosides (*aph(3’)-VI*). Upon transformation of the pNDMKP37 in *E. coli* DH5α, it conferred phenotypic resistance against ceftazidime, cefoxitin, and ampicillin/sulbactam, and intermediate sensitivity to ceftriaxone and imipenem. Moreover, the respective plasmid poses a great medical concern as it was responsible for the horizontal transmission of multidrug-resistant phenotypes to the transformant, particularly phenotypic resistance to carbapenems. New guidelines should be implemented for the rational use of broad-spectrum antibiotics, particularly carbapenems.

## Electronic supplementary material

Below is the link to the electronic supplementary material.

Supplementary Material 1

## Data Availability

All the data supporting the findings are included in the manuscript. The plasmid pNDMKP37 sequence project has been deposited in the GenBank under BioProject PRJNA878540, sample number SAMN30732688, and with the plasmid sequence accession number OK623716.1. The Sequence Read Archive (SRA) data are available from GenBank under the accession number SRR21492341. Acknowledgments:

## Abbreviations

AMR: Antimicrobial resistance
CLSI: Clinical and Laboratory Standards Institute
CR: Carbapenem resistance
CRE: Carbapenem-resistant
*Enterobacterales*, eCIM: EDTA-modified carbapenem inactivation method
ESBL: Extended-spectrum -lactamase
GNB: Gram-negative bacteria
MBL: Metallo--lactamase
mCIM: Modified carbapenem inactivation method
MDR: Multidrug-resistant
MGE: Mobile genetic element
NDM: New Delhi metallo--lactamase
ORF: Open reading frame
PCR: Polymerase chain reaction
WHO: World Health Organization
