# Supplementary material for: Transferable IncX3 plasmid harboring blaNDM-1, bleMBL, and aph(3’)-VI genes from Klebsiella pneumoniae conferring phenotypic carbapenem resistance in E. coli
Source: Mol Biol Rep. 2023 Apr 20;50(6):4945–53. doi: 10.1007/s11033-023-08401-9 (PMC10209314; doi:10.1007/s11033-023-08401-9)
Supplement: Supplementary file 1 — Supplementary Material 1 [file 11033_2023_8401_MOESM1_ESM.docx]

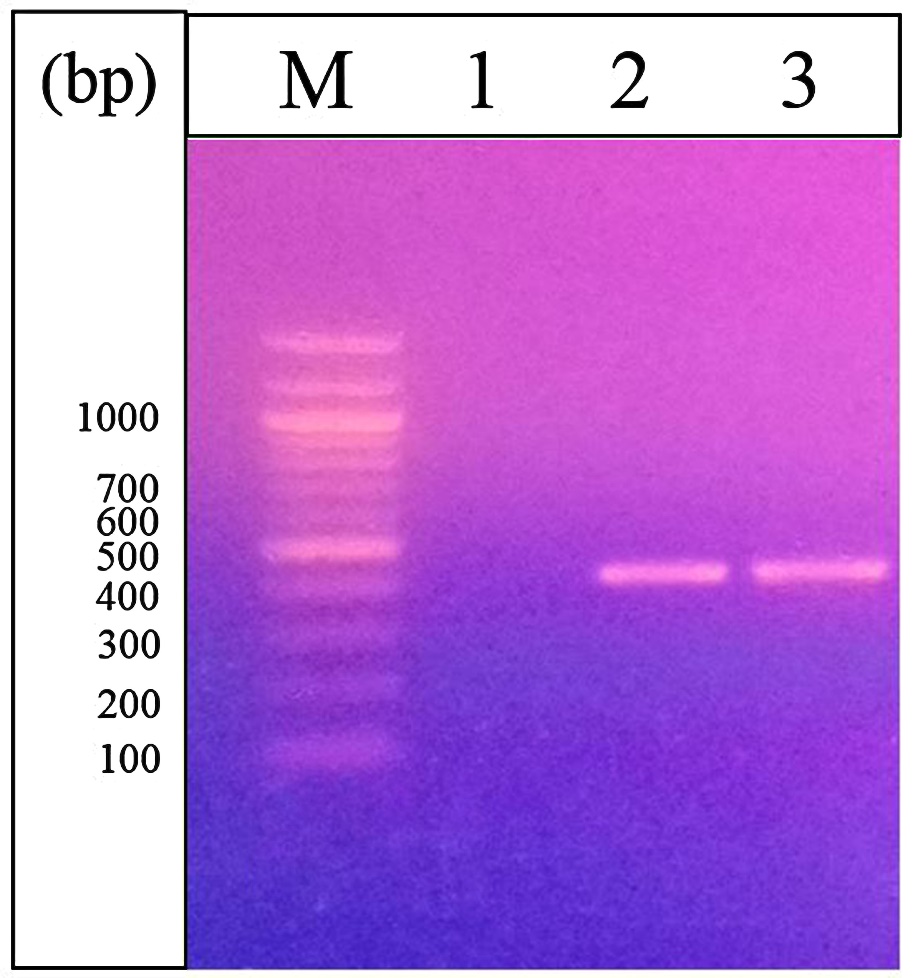


**Figure S1.** The results of agarose gel electrophoresis of the PCR amplification of *bla*_OXA-48_ gene (438 bp): Lane 1: negative control (untransformed *E. coli* DH5a); Lane 2: clinical *K. pneumoniae* isolate (37.AK); Lane 3: transformant (TS37.AK), lane M is a gene ruler 100 bp ladder (Thermo Fisher Scientific, Lithuania).


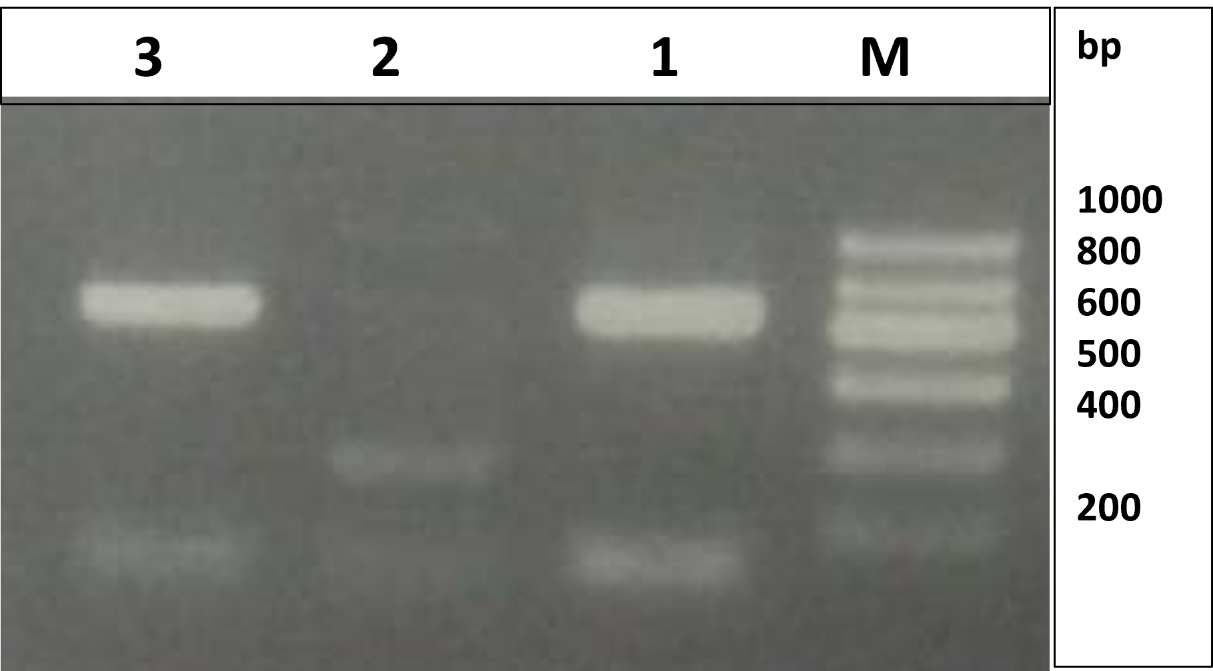


**Figure S2.** The results of agarose gel electrophoresis of the PCR amplification of *bla*_NDM_ gene (621 bp) of the plasmid extract of *K. pneumoniae* clinical isolate 37.AK (Lane 1); untransformed *E. coli* DH5α (lane 2, negative control); transformant *E. coli* DH5α, TS37.AK (Lane 3).Lane M, DNA Ladder 1kbp (Gene On Gmbh, Germany).
